# Preconceptional and Periconceptional Folic Acid Supplementation in the Visegrad Group Countries for the Prevention of Neural Tube Defects

**DOI:** 10.3390/nu17010126

**Published:** 2024-12-31

**Authors:** Vanda Rísová, Rami Saade, Vladimír Jakuš, Lívia Gajdošová, Ivan Varga, Jozef Záhumenský

**Affiliations:** 1 Institute of Histology and Embryology, Faculty of Medicine, Comenius University, 813 72 Bratislava, Slovakia; vanda.risova@fmed.uniba.sk (V.R.); ivan.varga@fmed.uniba.sk (I.V.); 2 2nd Department of Gynecology and Obstetrics, University Hospital Bratislava and Comenius University, 821 01 Bratislava, Slovakia; jozef.zahumensky@fmed.uniba.sk; 3 Institute of Medical Chemistry, Biochemistry and Clinical Biochemistry, Faculty of Medicine, Comenius University, 813 72 Bratislava, Slovakia; vladimir.jakus@fmed.uniba.sk (V.J.); livia.gajdosova@fmed.uniba.sk (L.G.)

**Keywords:** folic acid, neural tube defects, supplementation, polymorphisms, fortification, prevention, recommendations

## Abstract

Neural tube defects (NTDs) are malformations of the central nervous system that represent the second most common cause of congenital morbidity and mortality, following cardiovascular abnormalities. Maternal nutrition, particularly folic acid, a B vitamin, is crucial in the etiology of NTDs. FA plays a key role in DNA methylation, synthesis, and repair, acting as a cofactor in one-carbon transfer reactions essential for neural tube development. Randomized trials have shown that FA supplementation during preconceptional and periconceptional periods reduces the incidence of NTDs by nearly 80%. Consequently, it is recommended that all women of reproductive age take 400 µg of FA daily. Many countries have introduced FA fortification of staple foods to prevent NTDs, addressing the high rate of unplanned pregnancies. These policies have increased FA intake and decreased NTD incidence. Although the precise mechanisms by which FA protects against NTDs remain unclear, compelling evidence supports its efficacy in preventing most NTDs, leading to national recommendations for FA supplementation in women. This review focuses on preconceptional and periconceptional FA supplementation in the female population of the Visegrad Group countries (Slovakia, Czech Republic, Poland, and Hungary). Our findings emphasize the need for a comprehensive approach to NTDs, including FA supplementation programs, tailored counseling, and effective national-level policies.

## 1. Introduction

Neural tube defects (NTDs) constitute a group of congenital malformations resulting from the failure of the neurulation process, whereby the neural tube closes during the embryonic period, specifically between 21 and 28 days after fertilization. These disorders encompass a wide range of abnormalities affecting the structures of the developing central nervous system (brain and spinal cord), frequently involving the surrounding bone, muscle, and skin tissue [1]. These defects, which include conditions such as spina bifida and anencephaly, are among the most common congenital malformations worldwide [2]. The etiology of NTDs is multifactorial and is influenced by genetic (racial diversity, gender difference) [3], epigenetic (metabolic disorders of mother, proper nutrition—folic acid (FA), vitamin B12, trace elements) [4], non-genetic (geographical factors, socioeconomic status of parents) [5] and teratogenic (pesticides, arsenic, polycyclic aromatic hydrocarbons, antibiotics, anti-seizure medications, infections) [6,7] factors. Research suggests possible interactions between disrupted gene regulatory networks, environmental factors, and epigenetic regulation [8,9].

**Figure 1:**
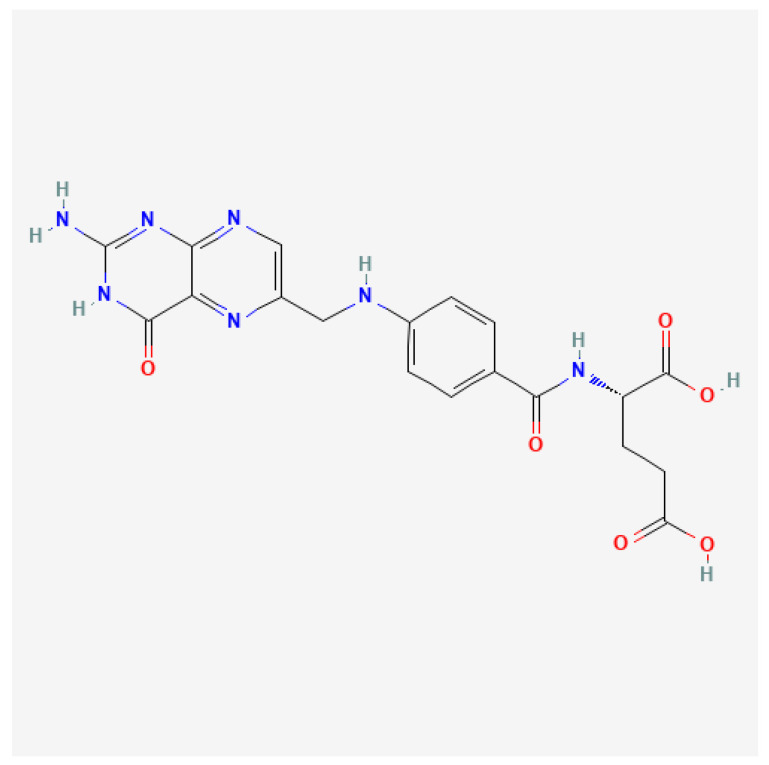
Molecular structure of folic acid [14].

Low preconceptional and periconceptional folate intake in women of reproductive age is one of the key risk factors for NTDs. FA, a synthetic form of folate, plays a crucial role in DNA synthesis, repair, and methylation, processes essential for normal neural development (Figure 1). Folate deficiency can cause DNA hypomethylation, block synthesis of 2′-deoxythymidine-5′-monophosphate and increase misincorporation of uracil. The exact prevalence of folate deficiency in women of reproductive age is not well-defined, but it is generally observed that in many lower-income countries, the prevalence may exceed 20%. In contrast, in higher-income countries, it is typically reported to be less than 5% [10]. The two most common genetic polymorphisms affecting the function of the key enzyme in folate metabolism, methylenetetrahydrofolate reductase (MTHFR), are C677T and A1298C. In the general population, approximately 60–70% of individuals carry at least one of the mentioned MTHFR gene variants. Among them, about 8.5% are homozygous for either the C677T or A1298C mutations, while 2.25% are compound heterozygous for both variants. Overall, around 10% of the population is either homozygous or compound heterozygous for the two mentioned polymorphisms [11,12]. The metabolic disruptions caused by mutations in MTHFR gene contribute to genomic instability, which increases the risk of NTDs. Currently, impaired de novo thymidylate biosynthesis (dTMP) and the accumulation of uracil in DNA are considered the only known metabolic risk factors for folate-sensitive NTDs [13].

NTDs represent a significant public health challenge, affecting thousands of pregnancies worldwide and requiring comprehensive prevention strategies [15]. These serious birth defects, occurring in early pregnancy, have profound implications for healthcare systems, families, and society, necessitating coordinated policy responses across national and regional levels. The prevention of NTDs through folic acid supplementation varies significantly across countries and regions. While some nations have implemented mandatory food fortification programs, others rely on voluntary approaches or educational initiatives. In the Visegrad Group countries, approaches range from comprehensive national programs to fragmented implementation strategies.

## 2. Historical Overview of FA Supplementation in the Visegrad Group Countries

The Visegrad Group (also known as the Visegrad Four or simply V4) was officially established on 15 February 1991, when the leaders of former Czechoslovakia, Poland and Hungary signed a declaration in Visegrad, Hungary. In this declaration, the Czech Republic, Slovakia, Hungary, and Poland pledged to collaborate in multiple fields of common national interests. The Visegrad Group countries, which share similar cultural, political, religious and intellectual values, aim to promote cooperation between the countries of Central Europe in various areas, including trade, nature conservation and international partnerships [16]. Although these countries are very similar in many aspects, our work aims to highlight the differences in policies regarding FA supplementation in the preconceptional and periconceptional periods among these countries.

In Hungary, early research conducted in the 1980s followed research methodology that explored the potential benefits of FA administration during pregnancy in preventing most malformations of the nervous system, especially NTDs [17]. In 1984, a clinical trial assessed the extent to which the use of FA or FA-containing multivitamins in the periconceptional period might contribute to a reduction in the incidence of early NTDs [18]. The results showed that a periconceptional multivitamin supplementation containing a daily dose of 800 µg of FA led to a significant reduction in the incidence of NTDs, as well as abnormalities of the urinary tract (particularly obstructive defects) and cardiovascular system malformations (especially ventricular septal defects) [19,20].

In the former Czechoslovakia, a study by Tolarová et al. examined the effects of periconceptional FA supplementation or FA-containing multivitamins on congenital anomalies, such as cleft lip with or without cleft palate [21].

In Poland, initatives launched in the late 1990s aimed to establish primary prevention programmes for NTDs, emphasizing the health benefits of FA supplementation for women of reproductive age [22]. Evidence demonstrated that the administration of FA resulted in a 70% reduction in the incidence of NTDs in newborns [23,24], as well as a decrease in other congenital defects, including abnormalities of the heart, limbs, urinary tract, digestive system, and orofacial clefts (Table 1) [25].

## 3. Neurulation and Genetic Predisposition to NTDs

**Figure 2:**
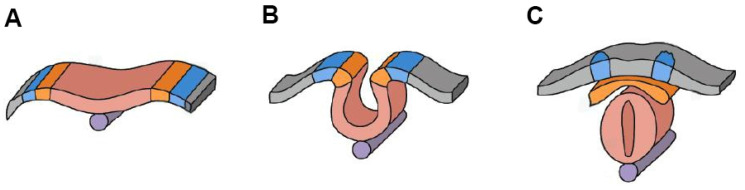
The three main stages of neurulation are illustrated sequentially (adapted from [33]). (**A**) Neural plate: A flat layer of ectoderm begins to bend as cells change their shape, laying the foundation for subsequent structures. (**B**) Neural folds: The folds elevate and converge toward the midline, facilitating the closure of the neural tube. (**C**) Neural tube: Once closed, this structure develops further into the central nervous system [33].

The process of neural tube formation and closure that results in the formation of the spinal cord and brain is called neurulation (Figure 2). This process takes place in the early stages of embryogenesis. Between the 23rd and 26th day of gestation, the neural tube definitively closes, and its lumen becomes the neural canal [29]. The process of neural tube closure depends on several biological processes, such as convergent neural plate extension, neural crest cells migration, and neuroepithelial apoptosis. Any abnormalities in these processes can interfere with the proper development and closure of the neural tube, which may promote the development of NTDs [30]. NTDs can be classified as “open NTDs”, where the nerve tissue is exposed and there is cerebrospinal fluid (CSF) leakage, or “closed NTDs”, where the nerve tissue is covered by tissue and there is no CSF leakage. Anencephaly and myelomeningocele (MMC) are the two most common forms of open NTDs. Anencephaly is a condition that arises as a result of the neural tube not closing in the cranial region, characterized by the absence of the skull, and is always fatal. Conversely, MMC results from the failure of the neural tube not closing in the spinal region. Closed NTDs are clinically categorized based on the presence (lipomyelocele, lipomyelomeningocele, meningocele, myelocystocele) or absence of a subcutaneous mass (dermal sinus, caudal regression, and segmental spinal dysgenesis) (Table 2) [31].

The genetic predisposition to NTDs arises from multiple metabolic and signaling pathways. Recent experimental studies have shown that the non-canonical signaling pathway (Wnt) and planar cell polarity (PCP) are directly involved in the processes of neurulation and neural tube closure. In addition, sonic hedgehog (Shh) signaling is critical for many aspects of early embryonic central nervous system development. Variants in PCP genes are increasingly implicated in NTD risk [32].

**Table 2:** Overview of causes and consequences of most common open and closed neural tube defects.

Neural Tube Defect	Characteristics	Causes	Consequences
Spina bifida	Incomplete closure of the spinal canal. Severe cases lead to paralysis of lower limbs and bladder/bowel dysfunction.	Genetic factors (e.g., MTHFR, FOLR1 SNPs), folate deficiency, infections during pregnancy, maternal diabetes, obesity.	Movement impairment, loss of sensation, incontinence, hydrocephalus, cognitive impairments [34].
Anencephaly	Absence of the brain and most of the skull.	Genetic factors, teratogenic substances (e.g., alcohol, drugs), maternal folate deficiency (increased risk with certain MTHFR SNPs), environmental toxins.	Often results in fetal death or death shortly after birth [35].
Encephalocele	External sac containing brain tissue on the head or neck.	Genetic factors (e.g., defects in early embryonic development), maternal folate deficiency, environmental factors.	Developmental delays, neurological abnormalities depending on sac size and location [36].
Myelomeningocele	Protrusion of meninges and part of the spinal cord from the back.	Incomplete closure of the neural tube during development, genetic factors (MTHFR polymorphisms), maternal folate deficiency, infections.	Severe paralysis, incontinence, pelvic organ dysfunction, cognitive and motor impairments [37].
Closed neural tube defects (lipomyelocele, lipomyelomeningocele, meningocele, myelocystocele)	Defects involving the accumulation of fatty tissue or abnormal spinal structures.	Less well-defined causes compared to open defects but may involve genetic predispositions (MTHFR and other folate-related SNPs), maternal folate deficiency, and early developmental issues.	Less severe but may lead to spinal deformities, mild neurological symptoms and developmental delays [31].

Author’s note: data provided in the table are incomplete; specific SNPs are not listed.

## 4. The Role of FA and One-Carbon Metabolism in the Etiology of NTDs

Recent research suggests that NTDs may be caused by multiple genetic mutations. Key mutations linked to NTDs include the MTHFR C677T polymorphism (locus 1p36.6), which impairs folate metabolism, and its prevalence varies between 10–30% in different populations [38]. Mutations in the FOLR1 (folate receptor 1) gene on chromosome 11q13.4 affect folate transport into cells, reducing folate availability to developing neural tissues. Although these mutations are rare, they have been linked to familial NTD cases [39]. Additionally, mutations in SLC25A32 (chromosome 8p11), which encodes a mitochondrial folate transporter are very rare, but have also been associated with NTDs, particularly when combined with folate deficiency [40]. The accumulation of these mutations, along with environmental factors like folate deficiency increases the risk of NTDs [41].

**Figure 3:**
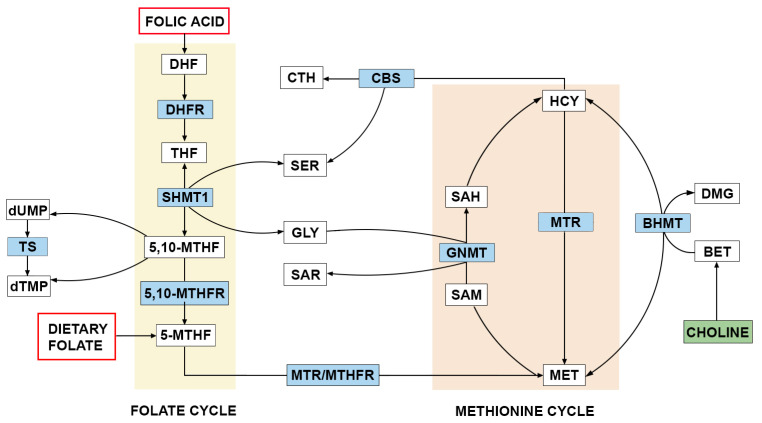
The role of folate and choline in one-carbon metabolism (adapted from [44]). 5,10-MTHFR, 5,10-methylenetetrahydrofolate reductase; 5,10-MTHF, 5,10-methylene-THF; 5-MTHF, 5-methylTHF; BET, betaine; BHMT, betaine-homocysteine S-methyltransferase; CBS, cystathionine β-synthase; CTH, cystathionine; DHF, dihydrofolate; DHFR, dihydrofolate reductase; DMG, dimethylglycine; dUMP, deoxyuridine monophosphate; dTMP, deoxythymidine monophosphate; GLY, glycine; GNMT, glycine N-methyltransferase; HCY, homocysteine; MET, methionine; MTR, methionine synthase; SAH, S-adenosylhomocysteine; SAM, S-adenosylmethionine; SAR, sarcosine; SER, serine; SHMT1, serine hydroxymethyltransferase 1; THF, tetrahydrofolate; TS, thymidylate synthase.

One-carbon metabolism (OCM) is a critical biochemical pathway that involves the transfer of one-carbon units for various cellular processes, including DNA synthesis and methylation (Figure 3). This pathway plays a vital role in fetal development, as it provides the necessary components for the formation of nucleotides and the regulation of gene expression, making it essential for neural tube closure and overall embryonic health. Mutations in genes encoding enzymes involved in OCM, such as MTHFR, methionine synthase (MTR), and thymidylate synthase (TS), are associated with an increased risk of NTDs [42]. Folate status can also be affected by polymorphisms in genes involved in folate metabolism. Folate metabolism promotes one-carbon transfer processes that contribute to the biosynthesis of purines and thymidine, the production of S-adenosylmethionine, the ubiquitous methyl donor required for the methylation of DNA, RNA, proteins, and lipids. These processes are carried out through linked reactions in the mitochondria and cytosol. During fetal development, the demand for one-carbon units is at its highest [43]. Choline is a critical nutrient also involved in OCM and serves as a methyl donor, playing an essential role in DNA methylation and cellular function. Adequate choline intake is associated with a reduced risk of NTDs. Given its interrelatedness with folate metabolism, ensuring sufficient choline levels during pregnancy is vital for optimal fetal development and may enhance the protective effects of folate against NTDs. Choline, as a one-carbon donor, plays a pivotal role in folate metabolism, facilitating its optimal utilisation and contributing to the prevention of the development of these developmental disorders.

NTDs can be caused by disruption of DNA synthesis, which is essential for cell proliferation [45]. Recent research has identified novel variants of the folate transporter (SLC19A1) and folate receptors (FOLR1, FOLR2, FOLR3) that impair folate metabolism and are associated with an increased risk of NTDs [46]. Cai et al. discovered additional polymorphisms in genes related to the folate metabolic pathway (MTHFR, MTHFD1, MTRR, RFC1) that act as risk factors for NTDs in mothers [47]. Reduced MTHFR activity leads to decreased levels of 5-methyltetrahydrofolate (5-MTHF) and increased levels of homocysteine (HCY), which may contribute to the production of reactive oxygen species and the development of oxidative stress [48]. However, research has shown that only 13% of NTDs can be attributed to the MTHFR C677T mutation, which suggests that the MTHFR C677T polymorphism alone may not be solely responsible for NTDs. It is possible that other factors such as gene-gene interactions, maternal-fetal interactions, or genetic-nutritional interactions may also play a role in the development of NTDs [49]. This is supported by a study by Behunova et al. in Slovak population, which found no significant association between polymorphisms C677T and A1298C of the MTHFR gene and NTDs [50].

## 5. Preconceptional and Periconceptional FA Supplementation in Visegrad Group Countries

In 1965, the hypothesis of a possible association between FA and a reduced incidence of NTDs was first proposed [51]. The first cohort-controlled studies demonstrated an embryoprotective effect of FA [52] and corroborated the findings that primary prevention of some major structural birth defects (NTDs, urinary and cardiovascular defects) by multivitamin supplementation with FA could be of significant public health importance [20]. Folate is essential for the proper functioning of various cellular processes, including amino acid metabolism, DNA methylation, protein and lipid synthesis, DNA replication, and cell division. During pregnancy, maternal folate requirement increases nearly fourfold to support the formation and cell division of the placenta and embryo, which are critical for fetal development [53]. FA requirement becomes even greater in lactating women than during pregnancy [54].

It is now common practice to prescribe FA to women in the preconceptual and periconceptual periods. Increased FA intake can be achieved in three different ways: by increasing the intake of folate-rich foods, supplementation in tablet form (FA-containing tablets or multivitamin preparations), and fortified foods (e.g., FA-fortified flour). The primary source of folate for humans is the diet. Folate occurs as polyglutamate in a variety of foods, including green leafy vegetables, legumes, liver, nuts, yeast, cereals, cereal sprouts, whole grain products, and citrus fruits (Table 3) [55]. However, supplements and fortified foods contain synthetic FA, which is found in the form of monoglutamate [56,57], an inactive form, which the human body is able to metabolize and must be converted to the active molecule 5-MTHF in the liver [58].

While adequate folate intake through a diverse diet is ideal, it can be challenging to achieve optimal levels, particularly during periods of increased demand, such as pregnancy. Lifestyle factors, dietary preferences, and socioeconomic disparities can influence dietary folate intake [61,62,63]. To address these challenges, a multifaceted approach is necessary.

While FA supplementation remains a cornerstone of prenatal care, it’s important to note that some individuals, particularly those with genetic variations in the MTHFR gene, may have difficulty converting folic acid into its active form. 5-MTHF is a more bioavailable form of folate, meaning it can be more readily used by the body. As such, it may offer additional benefits for certain populations, especially those with MTHFR polymorphisms. However, the optimal form of folate supplementation for individual women may vary, and it’s crucial to consult with a healthcare provider to determine the best approach [64,65].

Supplementation as a public health intervention, while beneficial, faces several challenges. One of the main challenges is that more than half of all pregnancies worldwide are unplanned, making it difficult to consume the necessary supplements before conception [66]. Large-scale FA fortification is considered the most successful and effective intervention program in reducing the incidence of NTDs and associated infant mortality. Fortification represents a population-level intervention that has multiple benefits. It can address nutritional deficiencies at the whole population level without the need for individual dietary changes, as well as address multiple nutritional deficiencies simultaneously, thereby increasing its effectiveness. Food fortification is a cost-effective strategy to improve nutritional status and prevent chronic diseases. It is implemented with respect to maximum tolerable daily intakes of micronutrients, minimizing the risks of excessive intake [67]. By July 2023, 69 countries have introduced mandatory FA fortification and 47 countries have established voluntary fortification. However, 77 countries have not yet introduced any FA fortification. Populations with mandatory FA fortification had 3- to 4-fold higher mean plasma folate levels than populations without any FA fortification [68]. De Wals et al. reported a decrease in the incidence of NTDs in seven Canadian provinces from 1.58 per 1000 births before fortification to 0.86 per 1000 births after fortification [69]. Recent estimates in the United States and Canada suggest that FA food fortification has provided additional intake of approximately 100 to 150 µg/day to women of reproductive age. Measurement of blood folate levels showed that red blood cell folate concentrations, a more robust indicator of folate status than plasma folate due to their ability to reflect long-term folate stores over weeks to months, increased by approximately 50% in each age group after the introduction of FA fortification [70]. In addition, data from the US National Birth Defects Prevention Study (1998–2003) suggest that the use of FA supplements during the periconceptional period no longer provides an additional reduction in the risk of NTDs. This suggests that fortification provides the necessary level of FA to prevent most folate-responsive NTDs [71]. Mandatory FA fortification programs may be more effective than supplementation programs in reducing the incidence of NTDs. This is because fortification provides FA to all food consumers, whereas periconceptional supplementation requires either planned pregnancy or continuous supplementation for all women of reproductive age and successful social interventions [72]. European Union (EU) countries including Poland [73], the Czech Republic, and Slovakia, are among the group of countries with recommended FA fortification [74]. In August 1998, a bread vitamin fortification programme was launched in Hungary, which resulted in the following nutritional composition of 200 g of bread: 400 µg FA, 25 µg vitamin B12, and 3600 µg vitamin B6 [75]. This strategy represents one of the earliest European attempts at population-wide FA fortification.

The food commissions of the EU countries have not yet agreed on the adoption of compulsory fortification, arguing that the impact of FA food fortification, whether positive or negative, on some selected diseases has not been sufficiently studied. Thus, FA fortification in EU countries remains only in the form of recommendations, while further research in this area is ongoing in parallel [76].

## 6. Comprehensive Strategies for NTDs Prevention

Effective NTDs prevention strategies and optimizing the nutritional status of women of reproductive age requires a comprehensive approach. This section presents evidence-based recommendations for FA supplementation and implementation strategies across the Visegrad Group countries. This includes consideration of individual factors such as recommended daily intake, optimal timing of FA supplementation, targeted counseling in the preconceptional and periconceptional periods, and strategic plans at the national level.

### 6.1. Recommended Daily Intake

Several factors related to digestion influence the absorption of vitamins from food. These factors include various medical conditions that can affect the body’s ability to absorb vitamins and other nutrients, either by directly impacting the digestive tract or by causing metabolic and nutrient distribution disorders. For instance, intestinal issues like Celiac disease [77], Crohn’s disease [78,79], or short bowel syndrome [80,81] can lead to malabsorption of vital nutrients, including folate. While a balanced diet, characterized by the consumption of a variety of nutrient-dense foods such as fruits, vegetables, lean proteins, and whole grains, generally provides essential nutrients necessary for good health, it often does not provide sufficient folate for women of childbearing age to meet the recommended intake levels required for NTD prevention [82]. Despite the consumption of folate-rich foods such as leafy greens, legumes, and fortified grains, studies have shown that dietary folate alone is frequently inadequate. This is due to several factors, including the heat sensitivity of folate, which is significantly reduced by cooking methods like boiling and frying [83]. The increasing consumption of processed foods, often low in folate content, further exacerbates this issue [84]. Therefore, folic acid supplementation is recommended to ensure that women receive the optimal dose of folate needed to support neural tube development in early pregnancy. The optimal dosage of FA remains a subject of debate. Health organizations such as the Centers for Disease Control and Prevention (CDC) and the World Health Organization (WHO) recommend that women who are planning to become pregnant or are already pregnant should take 400 µg of FA daily [85,86]. During the periconceptional period, it is recommended to increase the dose to 600 µg/day [63], while for women with a history of NTDs or other malformations, a dose of up to 800 µg/day, but not more than 1000 µg/day, is recommended due to possible adverse effects caused by excessive FA intake [87]. Dolin et al. emphasize the need to reconsider the recommended daily intake of FA to women at risk for recurrent NTD from 4000 µg to 1000 µg due to the reduced absorption rate and possible adverse health effects, including an increased risk of cleft palate, spontaneous abortion, impaired psychomotor development, and respiratory issues in children [88]. Furthermore, excessive doses of FA may increase the risk of complications in early pregnancy, as well as the development of insulin resistance, type 2 diabetes mellitus, and obesity in children [89]. The most prevalent complications include the potential masking of vitamin B12 deficiency, which poses severe risks for pregnant women with inadequate intake or impaired absorption of this vitamin. This deficiency can result in neurological impairment in the mother and potentially in the fetus if not properly identified and addressed [90]. On the other hand, Wald et al. asserted that a daily FA intake of 400 µg is insufficient to protect pregnant women from NTDs and proposed a daily FA intake of 500 µg, starting from the preconceptional period to enhance protection against the aforementioned defects [91]. The tolerable safe daily dose of FA for adults, including pregnant women, is 1000 µg per day [92]. While higher doses, up to 5000 µg per day, may be recommended for women in high-risk group of NTDs during early pregnancy, it is crucial to consult with a healthcare professional to determine the optimal dosage and duration of supplementation. Current consensus suggests that a dose of 5000 µg or higher is generally not justified, and a careful consideration of the benefits and risks is necessary when determining appropriate FA supplementation. Collectively, these findings underscore the importance of balancing FA intake to maximize NTD prevention while minimizing associated risks [93].

Following two groundbreaking studies that demonstrated the benefits of FA-containing supplements, medical and governmental organizations have published recommendations to promote the use of FA-containing supplements in the prevention of NTDs. The FA supplementation guidelines indicate considerable variability in the duration of preconceptional and periconceptional FA supplementation. Clinical studies confirm that at least 12 weeks of supplementation with 400 µg of FA before conception is required to achieve optimal erythrocyte folate levels for NTD prevention. The dose of preconceptional and periconceptional FA administration remains consistent in the European countries studied [94]. One of the more significant differences between the nations of the Visegrad Four concerns the recommended duration of FA supplementation before conception. Most European guidelines do not explicitly recommend a specific timeframe for initiating FA supplementation before conception. Crider et al. have shown that after 2–6 months of daily supplementation with 400 μg FA, erythrocyte folate levels reached 1000 nmol/L. Erythrocyte folate levels exceeding 1000 nmol/L are associated with a significantly reduced risk of NTDs. In non-supplemented individuals, erythrocyte folate concentrations below 500 nmol/L were linked to a significantly higher risk of NTDs, with an even higher risk associated with concentrations below 340 nmol/L [95]. Another discrepancy between countries is in the target group for whom FA supplementation is recommended. It is recommended that European guidelines move towards recommending FA for all women of childbearing age, or more specifically for all women at potential risk of pregnancy. This contrasts with the current recommendation of supplementation only to those women planning a pregnancy.

Due to the lack of approved recommended practices regarding FA supplementation in the preconceptional and periconceptional period in Slovakia, the Slovak Gynecological and Obstetric Society follows the recommendations of the American Society of Gynecologists and Obstetricians. A comparison of Slovak, Czech, Polish, and Hungarian recommendations on FA supplementation in the preconceptional, periconceptional, and lactation periods is presented in Table 4. While some countries, such as Poland, recommend higher doses (e.g., 5000 µg/day) for high-risk individuals, it’s important to balance the benefits and risks of such high doses. Higher doses may increase the risk of adverse effects, including those discussed earlier in the text. A personalized approach is crucial to ensure optimal supplementation while minimizing potential adverse effects.

### 6.2. Timing of Supplementation

The periconceptional period is the most vulnerable phase of embryonal development. During this period, adequate folate levels are particularly important to support the formation of the neural tube, often before a woman is aware she is pregnant. For this reason, women of childbearing age, especially those planning to become pregnant, are encouraged to ensure sufficient folate intake through a combination of a healthy diet and dietary supplements. While a diet rich in folate-containing foods should be a staple during pregnancy, it can be challenging for women to meet the recommended folate intake solely through food. Consequently, supplementation is often advised to guarantee adequate folate levels, particularly during the preconception period and the initial months of pregnancy. This proactive approach helps to address potential dietary deficiencies and ensures that women receive the necessary nutrients to support healthy embryonic development. It may take up to 20 weeks for a pregnant woman to reach the optimal erythrocyte folate level (1050–1340 nmol/L) needed to reduce the risk of NTDs when supplementing 400 µg of FA daily. For this reason, it is advisable to start supplementation 5 to 6 months before conception [96]. When these optimal levels are reached, the risk of NTDs is approximately 4.5 cases per 10,000 births [58]. Dong et al. suggest that the optimal time to start FA supplementation is 1.5 months before pregnancy, with an acceptable range of 1.1–1.9 months. Women in this study who supplemented with FA during the mentioned period had a 1.52% risk of congenital malformations. The recommended duration of FA supplementation is 4 (3.7–4.4) months [97]. Determining the optimal time and duration of FA supplementation is critical for maximum protection and prevention of side effects that result from excessive FA supplementation [98].

Prenatal care in gynecological outpatient clinics usually does not begin until after the 7th week of pregnancy, which delays the start of FA supplementation. Some countries, such as Vietnam, recommend starting FA supplementation from the first antenatal visit, which usually takes place at 16 weeks of pregnancy [99]. Nilsen et al. found that only 48.6% of Italian women who sought preconception healthcare and planned to become pregnant were taking FA before pregnancy [100]. This suggests that Italian women start taking FA supplements too late to prevent NTDs. China has one of the highest NTD prevalence rates in the world, with inadequate dietary folate intake thought to be the main cause. According to Ren’s study, less than a quarter of women took FA before becoming pregnant, leaving three-quarters of fetuses at risk during the critical period of neural tube formation [101].

### 6.3. Preconception Counselling

The lack of uptake of FA in women happens due to a lack of knowledge and awareness of its protective effect against NTDs. For women, who are planning to become pregnant, antenatal counselling is a valuable strategy [102]. Health care providers play a key role in providing preconception counselling to women. Counselling should include a comprehensive discussion of the benefits of FA supplementation, the optimal timing and duration of supplementation, as well as pregnancy planning and addressing specific risk factors a woman may have. A thorough nutritional assessment to identify the risk of folate deficiency should also be an essential part of the evaluation. This assessment should identify and evaluate dietary and lifestyle habits (e.g., high alcohol intake [103], vegetarianism, veganism [104], smoking [105], excessive caffeine consumption [106]) that may affect folate absorption and metabolism. The woman’s diet should be analyzed to determine whether it contains sufficient natural sources of folate, such as dark green leafy vegetables, legumes, and citrus fruits. Additionally, factors such as gastrointestinal diseases (e.g., celiac disease, Crohn’s disease) that may impact folate absorption should also be considered [107].

### 6.4. National Level Strategic Plan

Many countries have implemented campaigns to encourage women in the preconceptional and periconceptional periods to take regular FA supplementation and thus raise awareness of the importance of FA intake. Clarification of international recommendations, fortification of flour, as well as other foods could increase the effectiveness of folate supplementation at the national level.

National level strategies vary in their approach and policy implementation. The Polish government introduced the programme called “Primary NTD prophylaxis” in 1998, recommending a daily FA intake of 400 µg for women of childbearing age. The aim of the programme was to promote awareness of FA and its relationship to NTDs, to influence attitudes, and to encourage appropriate behaviors in women regarding FA supplementation. It was implemented in health facilities providing services to pregnant women, integrated into health education projects, and disseminated through mass media [73].

In 2010, the Czech Republic launched the “Think of Me Before I’m Born” programme, focusing on the issue of FA and its recommended supplementation. The programme encouraged women who were trying to conceive or might become pregnant to take dietary supplements containing FA [108].

While national level strategies for FA supplementation are still under consideration in Slovakia, recent efforts have focused on raising awareness of the critical role of FA intake in the prevention of NTDs. Educational initiatives targeting women of childbearing age, as well as partnerships with healthcare providers aim to increase public knowledge of the benefits of FA intake, particularly during the preconceptional period. Continued efforts, including potential fortification policies and further public health campaigns will enhance the effectiveness of FA supplementation at the national level.

## 7. Conclusions

As FA fortification of foods is still not mandatory in Europe, there is a pressing need for evidence-based guidelines for FA supplementation before conception and during pregnancy. Regional discrepancy in the prevalence of NTDs may be influenced by geographical differences in dietary folate intake and the distribution of the MTHFR C677T polymorphism in the population. Preconceptional and periconceptional FA supplementation, as well as nationwide food fortification programs, have successfully prevented thousands of NTDs. However, given the genetic complexity of these defects and their persistent interactions with environmental factors, complete prevention of NTDs remains an unattainable goal. The optimal approach is early primary prevention, exemplified by the population-wide use of FA supplements in pregnancy planning.

This review compares and provides practical guidelines and policy recommendations for implementing effective FA supplementation strategies in the Visegrad Group countries. Although research on FA supplementation in the preconceptional and periconceptional periods dates back to 1984, the results obtained from randomized trials have not been adequately implemented in practice, even after 40 years. It is essential that each country has clearly defined clinical practice guidelines, based on current evidence-based medicine, to guide the provision of health care, both in major cities and in more remote urban and rural areas. Historically and socio-culturally, there are many similarities among the Visegrad Group countries, but still significant differences in health care delivery can be found, as in the case of FA supplementation recommendations. As shown in Table 4, there are considerable differences in folate intake during pregnancy among these countries. In Slovakia, there is currently no clear standard guideline on FA supplementation in the pre- and periconceptional periods issued by the Slovak Society of Gynaecology and Obstetrics. In practice, doctors follow the recommendations of foreign organizations, which are not uniform and differ in the recommended dosage, as well as the period when FA should be taken as dietary supplements. Considering the prevalence of MTHFR gene mutations in the Slovak population, it is necessary to develop a standardized procedure and consider the introduction of food fortification with FA, either voluntarily or obligatorily, similar to some other countries. In contrast to Slovakia, the Czech Republic, and Hungary, the Polish Society of Gynaecologists and Obstetricians has developed comprehensive recommendations for FA and other vitamin supplementation, serving as a model for other countries in the Visegrad region.

FA supplementation cannot guarantee 100% prevention of NTDs because up to one-third of these defects are FA-resistant. Currently, there is a strong emphasis on personalized medicine, the search for alternative preventive strategies, and the exploration of new biomarkers using omics technologies supported by artificial intelligence. Precision medicine strategies that harness the power of human genomics and advanced tools for assessing genetic risk factors will be essential for future prevention initiatives.

## Figures and Tables

**Table 1:** Association of folate intake deficiency with congenital disorders.

Congenital Disorder	Influence of Folate Intake
Neural tube defects	Folate deficiency in pregnancy increases risk of closed and open NTDs [18].
Heart defects	Research suggests that folate deficiency during pregnancy may be associated with an increased risk of congenital heart defects (non-syndromic septal, conotruncal, right or left-sided obstructive heart defect) [26].
Cleft lip and palate	Some studies suggest that sufficient folate intake may reduce the risk of cleft lip and palate in newborns [27].
Limb deformities	Folate is important for proper limb development; its deficiency may be associated with a variety of limb reduction defects [28].

**Table 3:** List of food examples and their folate and choline content [59,60].

Food	Folate Content (µg/100 g)	Choline Content (mg/100 g)
	High folate content (≥100 µg/100 g)	
Beef liver (raw)	290	333
Spinach (raw)	194	19.3
Lentils (cooked)	181	32.7
Chickpeas (cooked)	172	42.8
Asparagus (cooked)	149	26.1
	Moderate folate content (30–99 µg/100 g)	
Avocado (raw)	89	14.2
Peas (raw)	65	28.4
Broccoli (raw)	63	18.7
Kimchi	52	15.5
Red bell pepper (raw)	47	5.6
Eggs (whole, cooked)	44	294
Mango (raw)	43	7.6
Bread (whole wheat)	42	27.2
	Low folate content (<30 µg/100 g)	
Salmon (Atlantic, farmed, raw)	26	78.5
Orange (raw)	25	8.4
Sauerkraut (fermented cabbage)	23	10.4
Carrots (raw)	19	8.8
Tofu	15	28.8
Potatoes (baked)	9	14.5
Chicken breast (cooked)	4	35
White rice (cooked)	1	2.1

**Table 4:** Comparison of Slovak, Czech, Polish, and Hungarian recommendations for folic acid supplementation during preconceptional, periconceptional and lactation period.

Country	Recommending Society	Folic Acid Dosage			Common Recommendations
		Low Risk *	Intermediate Risk **	High Risk ***	
Slovakia	The American Society of Gynaecologists and Obstetricians [54]	400 µg/day 2–3 months prior to pregnancy and throughout the 1st trimester; 600 µg/day is recommended during the 2nd and 3rd trimester and throughout lactation	1000 µg/day 3 months prior to pregnancy and throughout the 1st trimester	4000 µg/day 3 months prior to pregnancy and the entire 1st trimester	A diet rich in folate is recommended for women of reproductive age. Vitamin B12 supplementation is recommended along with folate. The dosage of folic acid is based on the risk of NTDs.
Czechia	The Czech Society of Gynaecologists and Obstetricians [51]	400–800 µg/day one month prior to pregnancy and throughout the 1st trimester	4000 µg/day in case of previous NTD pregnancy, BMI > 30, or genetic mutations in folate metabolism	N/A	
Poland	The Polish Society of Gynaecologists and Obstetricians [89]	400 µg/day 3 months prior to pregnancy, during pregnancy, and lactation	800 µg/day 3 months prior to pregnancy, during pregnancy, and lactation in case of pre-existing type 1 or 2 diabetes mellitus, use of antiepileptic drugs, or bariatric surgery	5000 µg/day 3 months prior to pregnancy and throughout 1st trimester, 800 µg/day throughout 2nd and 3rd trimester and lactation	
Hungary	The National Institute for Health Promotion in Hungary and The National Council of Hungarian Gynaecologists [94]	400 µg/day 3 months prior to and during pregnancy	N/A	N/A	

* Low Risk: Women without a history of NTDs or other risk factors. ** Intermediate Risk: Women with conditions such as obesity (BMI > 30), previous NTD-affected pregnancies, or genetic mutations affecting folate metabolism (e.g., MTHFR mutations). *** High Risk: Women with pre-existing medical conditions such as type 1 or type 2 diabetes, use of antiepileptic drugs, or a history of bariatric surgery.

## Data Availability

No new data were created or analyzed in this study. Data sharing is not applicable to this article.
